# Automation of Multi-Class Microscopy Image Classification Based on the Microorganisms Taxonomic Features Extraction

**DOI:** 10.3390/jimaging11060201

**Published:** 2025-06-18

**Authors:** Aleksei Samarin, Alexander Savelev, Aleksei Toropov, Aleksandra Dozortseva, Egor Kotenko, Artem Nazarenko, Alexander Motyko, Galiya Narova, Elena Mikhailova, Valentin Malykh

**Affiliations:** 1 Higher School of Digital Culture, ITMO University, St. Petersburg 197101, Russia; 2 Faculty of Radio Engineering and Telecommunications, St. Petersburg Electrotechnical University “LETI”, St. Petersburg 197022, Russia; 3 Department of Microbiological Synthesis Technology, St. Petersburg State Institute of Technology, St. Petersburg 190013, Russia; 4 Information Systems Department, International IT University, Almaty 050000, Kazakhstan

**Keywords:** biomedical image processing, multi-class classification, microbial recognition, taxonomic features extraction, filter-based preprocessing

## Abstract

This study presents a unified low-parameter approach to multi-class classification of microorganisms (micrococci, diplococci, streptococci, and bacilli) based on automated machine learning. The method is designed to produce interpretable taxonomic descriptors through analysis of the external geometric characteristics of microorganisms, including cell shape, colony organization, and dynamic behavior in unfixed microscopic scenes. A key advantage of the proposed approach is its lightweight nature: the resulting models have significantly fewer parameters than deep learning-based alternatives, enabling fast inference even on standard CPU hardware. An annotated dataset containing images of four bacterial types obtained under conditions simulating real clinical trials has been developed and published to validate the method. The results (Precision = 0.910, Recall = 0.901, and F1-score = 0.905) confirm the effectiveness of the proposed method for biomedical diagnostic tasks, especially in settings with limited computational resources and a need for feature interpretability. Our approach demonstrates performance comparable to state-of-the-art methods while offering superior efficiency and lightweight design due to its significantly reduced number of parameters.

## 1. Introduction

Infectious diseases remain one of the leading causes of mortality and economic loss on a global scale. According to the WHO [1], in 2019, bacterial infections, including pneumonia, meningitis, and sepsis, killed more than 7 million people, and the growth of antibiotic resistance threatens to negate the achievements of modern medicine [2,3]. The COVID-19 [4] pandemic vividly demonstrated humanity’s vulnerability to pathogens: bacterial co-infections significantly worsened the prognosis in patients with SARS-CoV-2 [5], and errors in diagnosis aggravated the burden on healthcare systems [6,7]. In this context, the rapid and accurate identification of microorganisms is becoming not just a scientific task but a critical tool for saving lives.

Bacteria surround humans everywhere: they are involved in digestion, drug production, food fermentation, and maintaining ecological balance [8]. However, among thousands of bacterial species, dangerous pathogens can cause serious diseases. For example, Lactobacillus promotes intestinal health, while Salmonella leads to food poisoning [9]. This diversity requires a clear distinction between beneficial and harmful microorganisms, especially in clinical practice.

**Figure 1:**
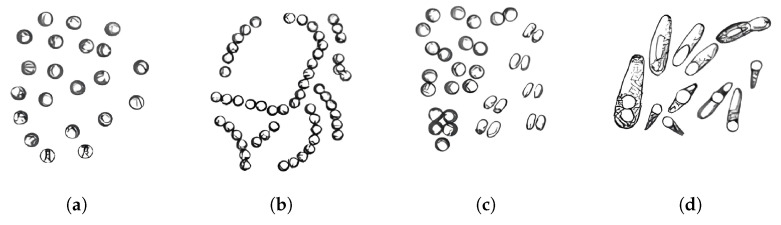
Bacterial cell morphology: (**a**) micrococci; (**b**) streptococci; (**c**) diplococci; (**d**) bacilli.

Biologists traditionally classify bacteria based on morphology [10] (cell shape, size, colony structure, etc.) and biochemical properties [11]. For instance, cocci [12] (spherical), bacilli [13] (rod-shaped), and spirilla [14] (spiral-shaped) can be visually distinguished, as shown in Figure 1, yet even within a single group, there exist so-called “look-alike” species. For example, Streptococcus pneumoniae [15] (diplococcus) and Enterococcus faecalis [16] (chains of cocci) can be misidentified during manual microscopy [17]. Moreover, some bacteria alter their shape and metabolism in response to environmental conditions, further complicating their identification even for experienced specialists [18].

Among bacteria requiring close attention, four groups stand out due to their clinical and epidemiological significance:

1. Micrococci [19]: These are facultatively pathogenic Gram-positive cocci that can cause opportunistic infections, particularly in immunocompromised patients. While generally considered low-virulence organisms, their ability to exploit weakened host defenses makes them a notable concern in healthcare settings [20].
2. Diplococci [21]: These pathogens are responsible for severe diseases such as pneumonia and meningitis. Notably, Streptococcus pneumoniae alone accounts for approximately 15% of childhood mortality in low-income countries, highlighting its devastating impact on vulnerable populations.
3. Streptococci [22]: This genus includes pathogens that cause a wide range of diseases, from pharyngitis and scarlet fever to severe post-infectious complications such as rheumatic fever. Globally, streptococcal infections affect over 600 million people annually, underscoring their pervasive public health burden [23].
4. Bacilli [24]: This group encompasses species such as B. anthracis, the causative agent of anthrax, and B. cereus, a common culprit in foodborne illnesses. While B. anthracis poses significant biosecurity risks due to its potential use as a biological weapon, B. cereus is a frequent cause of gastroenteritis, particularly in improperly stored food. Both species exemplify the dual threat posed by bacilli to both individual health and broader biosecurity [25].

Given the high clinical and epidemiological impact of these bacterial groups, accurate identification is critical for effective treatment and infection control. While conventional diagnostic methods, such as Gram staining and culture-based techniques, remain widely used, they have limitations, including long processing times and potential misidentifications due to morphological similarities.

Recent advancements in molecular diagnostics have significantly improved bacterial identification. Methods such as MALDI-TOF mass spectrometry [26], polymerase chain reaction (PCR) [27], and next-generation sequencing (NGS) [28] enable rapid and precise differentiation of closely related bacterial species. These technologies allow clinicians to identify pathogens within hours rather than days, leading to faster and more targeted antibiotic therapy. However, in resource-limited settings, where bacterial infections are most prevalent, accessibility to these advanced methods remains a challenge. Developing cost-effective, rapid diagnostic solutions is, therefore, a key priority for global health initiatives.

## 2. Related Work

The automatic classification of microorganisms has been extensively studied in biomedical image processing, with a primary focus on deep learning techniques, feature extraction methods, and automated machine learning (AutoML) [29,30,31] approaches. Traditional microbiological diagnostics, which rely on manual microscopy and biochemical assays, have long been the gold standard for bacterial identification but suffer from subjectivity, long processing times, and potential human errors [11,32]. Recent advancements in machine learning have enabled the development of automated methods that enhance diagnostic accuracy and efficiency.

Currently, there are many publicly available datasets for the task of classifying microorganisms in microscope images. At the same time, for these datasets [33], there are quality metrics for a wide range of models, such as convolutional networks [34] or transformers [35]. However, it should be noted that most of these datasets [36] contain images of a fixed microscopic scene, in which the microorganisms are immobilized and the scene itself has high contrast compared to the image of a non-fixed substance. In our work, we focus precisely on the study of microscopic scenes, which prompted us to collect and annotate our own dataset presented in this article.

Early efforts in bacterial classification employed handcrafted feature extraction techniques, such as texture analysis [37], shape descriptors [38], and statistical models [39]. The mentioned approaches explore the use of statistical image analysis and machine learning to classify bacteria based on morphological and textural features extracted from microscopic images. The authors developed computational models to automate bacterial identification, reducing reliance on manual analysis. This method’s approach enhances accuracy and scalability, providing a robust framework for high-throughput analysis. Regarding its limitations, the performance depends on image quality, and the method requires large labeled datasets, while some models lack interpretability. The obtained results highlight the potential of data-driven bacterial identification but also underscore key challenges in reproducibility and real-world applicability.

These methods allowed for some degree of automation but lacked the adaptability of modern deep-learning models. The advent of convolutional neural networks (CNNs) revolutionized bacterial image classification by enabling end-to-end feature learning directly from images [40,41]. Several studies have demonstrated the effectiveness of CNNs [42,43] in recognizing various bacterial morphologies, including cocci, bacilli, and spirochetes, under different microscopy conditions [44,45].

However, deep learning approaches have significant limitations in microbiology. First, CNNs require large, well-annotated datasets to achieve high accuracy, which can be challenging to obtain in specialized domains [46]. Second, biomedical images often exhibit domain-specific challenges, such as low contrast, noise, and motion artifacts, making deep learning models prone to overfitting or misclassification [47]. Third, the interpretability of CNNs remains a concern, as black-box models hinder the trustworthiness and clinical adoption of AI-based diagnostic systems [48].

Hybrid approaches combining deep learning with traditional feature engineering have been proposed to address these challenges. Methods leveraging deep feature extraction followed by classical classifiers (e.g., Support Vector Machines and Random Forests) have shown promise in improving classification performance while retaining some level of interpretability [49,50]. Additionally, transformer-based architectures, such as Vision Transformers (ViTs), have recently gained traction in medical imaging because they capture long-range dependencies in visual data [51]. However, ViTs require large-scale datasets and computational resources, limiting their feasibility in microbiology [52].

AutoML represents a promising alternative by automating machine learning pipeline selection, optimization, and evaluation. Recent studies have explored AutoML for various biomedical applications, including histopathological image classification [53], tumor detection [54], and genomic analysis [55]. AutoML has been utilized in bacterial classification to optimize feature extraction and model selection, reducing the need for extensive hyperparameter tuning and domain expertise [56]. The ability of AutoML to generate interpretable feature spaces by leveraging geometric and morphological descriptors makes it particularly suitable for microbiological diagnostics [57].

In this study, we apply AutoML to the multi-class classification of microorganisms, including micrococci, diplococci, streptococci, and bacilli. Our approach integrates feature space generation based on external geometric properties with an automated pipeline for classifier selection and optimization. Compared to existing deep learning-based methods, our technique enhances interpretability while maintaining high classification performance under challenging microscopy conditions. Notably, the resulting models have few parameters and demonstrate fast inference even on standard CPU hardware, making the approach suitable for real-time or resource-constrained biomedical applications. The results demonstrate that AutoML can be a robust and scalable tool for biomedical diagnostics, addressing the growing need for rapid and reliable pathogen identification.

## 3. Problem Statement

The classification of microorganisms is traditionally based on microscopic analysis, where experts identify bacteria by their shape, size, and cell organization. However, this approach has two serious limitations. Firstly, it is subject to subjectivity, since the interpretation of morphological features depends on the experience of the laboratory technician. For example, diplococci may be mistaken for single cocci and streptococci for image artefacts [20]. Secondly, manual analysis requires considerable time. In the context of epidemics, the promptness of diagnosis becomes a critical factor: a 24-hour delay in the identification of Streptococcus pneumoniae increases the risk of death by sepsis by 18% [58].

Automation of the classification process based on computer vision methods seems to be a promising solution that can eliminate subjectivity and increase the speed of analysis. However, modern neural network approaches, in particular convolutional neural networks (CNNs), face several difficulties. First, they require large amounts of host data, which are often unavailable in microbiology. Secondly, biomedical images have specific features such as low contrast, blurred object boundaries, and motion artifacts in unfixed samples, which make it difficult to classify them [59] accurately.

In this study, we solve the problems of multi-class classification of microorganisms by using the AutoML method to construct a feature space and optimize classifiers automatically. There are six classes in our dataset: micrococci, diplococci, streptococci, bacilli, other microorganisms, and areas without bacteria. The proposed method combines the analysis of the geometric characteristics of bacterial cells with the capabilities of AutoML, which makes it possible to achieve high classification accuracy and adaptability to difficult microscopy conditions. The main goal of the work is to develop an effective and interpretable tool for the diagnosis of bacterial infections, which can be used both in well-equipped laboratories and resource-limited conditions.

## 4. Materials and Methods

**Figure 2:**
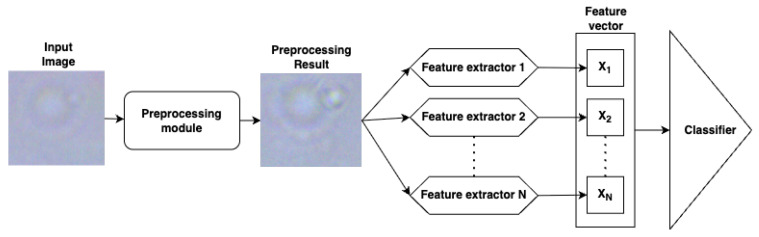
The general structure of the proposed classification model using the image of diplococci as an example.

Our method is built upon a sequential architecture that progressively transforms raw microscopic images into meaningful predictions. The pipeline begins with image preprocessing aimed at enhancing visual clarity and ensuring consistency across samples. This preparatory step lays the foundation for robust analysis by minimizing noise and standardizing input quality. Next, the system performs targeted extraction of informative geometric and morphological features, capturing the essential structural traits of microorganisms. These primary descriptors are then automatically expanded into higher-order representations, enriching the feature space with more abstract and discriminative patterns. Finally, a classifier processes the resulting feature vectors, yielding accurate microorganism identification. An overview of the full classification workflow is presented in Figure 2.

### 4.1. Image Preprocessing

Since unfixed microscopic scenes have poor contrast, as well as artifacts associated with blurriness and variable brightness, and it was difficult to select parameters for the corresponding correction filters based on any rules, we used a lightweight neural network model based on the LFIEM (UniFi modification) [60,61] neural network architecture, which selects parameters in such a way as to configure preprocessing filters to achieve maximum quality metrics. The model used showed high results [61] on common color correction datasets and also contains a multiple of fewer parameters compared to analogs [60,61]. The experimental data also confirmed the feasibility of its use to improve the quality of our classifier.

The preprocessing approach presented in [60] was employed with several refinements, as outlined below. The modified structure of the corrective transformation process is depicted in the diagram that follows.

(1) $$I_{e}=I_{o}+\sum_{i=1}^{n}f_{i}\left(I_{o},h_{i}\left(I_{so}\right)\right).$$

This architecture consists of multiple independent processing units, with their quantity determined by the selected filters. Each unit *i* operates on a downsampled version of the original image $I_{so}$ through a parameter generator $h_{i}$, which derives the corresponding filter parameters $p_{i}$ for $f_{i}$. These filters are then individually applied to the initial image $I_{o}$, and the final enhanced image is obtained by combining the original input with the outputs of the filtering operations.

To mitigate common challenges in microscopic scene images, such as blurring, low contrast, and insufficient sharpness, we employed specialized filters tailored for corrective transformations.

The sharp filter is defined using the following auxiliary formula:

(2) $$I_{out}=I_{in}⊛\frac{1}{\nu }\left(K+M\cdot q\right),$$

where *K* represents the filter kernel matrix, *M* denotes a mapping matrix of the same dimensions as *K*, and ν is the sum of all elements in (K+M·q), ensuring proper normalization of the kernel matrix. This transformation is independently applied to the red, green, and blue color channels, with each controlled by a distinct trainable parameter. Therefore, the parameters that define the sharp filter modification are as follows:

$$\begin{array}{cc} K & =\left(\begin{array}{ccccc} 1 & 4 & 6 & 4 & 1\\ 4 & 16 & 24 & 16 & 4\\ 6 & 24 & -476 & 24 & 6\\ 4 & 16 & 24 & 16 & 4\\ 1 & 4 & 6 & 4 & 1 \end{array}\right),\\ M & =\left(\begin{array}{ccccc} 0.8 & 0.8 & 0.8 & 0.8 & 0.8\\ 0.8 & 0.9 & 0.9 & 0.9 & 0.8\\ 0.8 & 0.9 & 1 & 0.9 & 0.8\\ 0.8 & 0.9 & 0.9 & 0.9 & 0.8\\ 0.8 & 0.8 & 0.8 & 0.8 & 0.8 \end{array}\right). \end{array}$$

The automatic contrast correction is achieved by adjusting the parameter p∈[−1,1], which governs the transformation applied to each pixel in the input image. As a result, the original image undergoes the following mapping:

(3) $$I_{out}\left[x,y\right]=\left\{\begin{array}{cc} \left(I_{in}\left[x,y\right]-0.5\right)\cdot \frac{1}{1-r}, & \mathrm{if}\;r>0\\ (I_{in}\left[x,y\right]-0.5)\cdot \left(1-r\right), & \mathrm{otherwise}. \end{array}\right.$$

It is important to emphasize that global exposure adjustments are essential due to the significant variations in illumination conditions observed during microscopic imaging.

The following image transformation performs automatic exposure correction:

(4) $$I_{out}\left[x,y\right]=I_{in}\left[x,y\right]\cdot 2^{t}.$$

By reducing the number of transformations, we successfully integrated predictors for all parameters into a unified neural network encoder.

**Figure 3:**
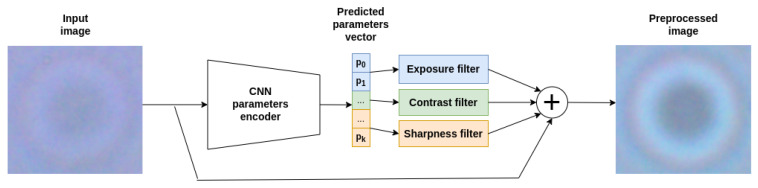
Image preprocessing module structure using the image of micrococci as an example.

Thus, the structural design of the preprocessing module is illustrated in Figure 3.

To train our neural network parameter encoder, we manually selected 2000 good-quality microorganism images and applied fixed-value filters to emulate bad ones. We then trained a hybrid preprocessing scheme to reconstruct good images, following the procedure described in the original LFIEM paper.

### 4.2. Contour Primitives Determination

**Figure 4:**
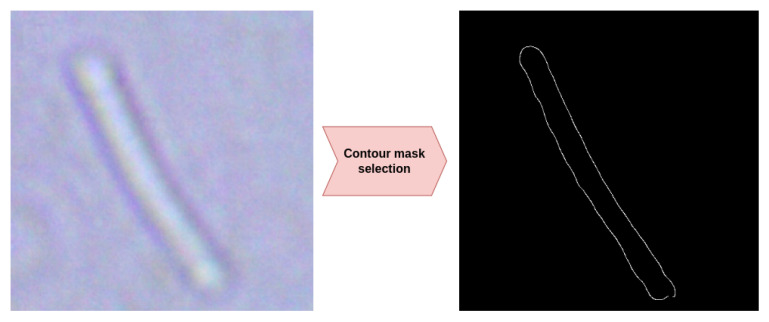
Visualization of the result of applying the automatic contour extraction procedure we used using the example of an image of a bacillus obtained from a microscope.

To achieve precise bacterial segmentation within bounding boxes, we employed advanced active contour models combined with morphological optimization. Contour selection (Figure 4) is used for further calculation of the characteristics of interest to us related to the shape of the objects under study (for example, when studying area characteristics and properties of segmentation masks). The pipeline begins with Selective Adaptive Thresholding–Active Contours (SAT-AC) [62], which integrates region-based energy terms with edge-driven forces for robust boundary detection in low-contrast microscopy images. Gradient enhancement is performed using anisotropic multi-scale Gabor filters [63], improving sensitivity to faint bacterial edges while suppressing noise. For shape preservation, curvature-constrained morphological smoothing [64] is applied, maintaining delicate structures (e.g., flagella or diplococci chains). It should also be noted that in our pipeline, we have minimized the risk of artifacts when selecting contours using the previous step of image preprocessing (getting rid of blur, increasing contrast, and correcting artifacts related to brightness). For each pipeline we studied, we selected the optimal parameters for these transformations using grid search and selected an appropriate configuration according to overall classification performance metrics maximization.

Using the contour characteristic, we calculated the statistical values describing the shapes of microorganisms, as is given further in the text of the article.

### 4.3. Automated Feature Generation

Within the framework of the presented methodology, we embarked on an in-depth exploration of the multi-faceted traits demonstrated by microorganisms, as revealed through advanced computational microscopy. At the outset, our procedure emphasizes the deliberate extraction of a wide array of quantifiable descriptors characterizing the biological sample. To streamline both interpretation and subsequent processing stages, these descriptors, along with the analytical techniques employed, are systematically organized into three foundational categories. This tripartite structure not only brings clarity to the complexity of the data but also lays the groundwork for constructing resilient and well-generalized analytical frameworks capable of supporting reliable classification and interpretation.

The first category. To initiate the analysis, we extract a range of prominent geometric descriptors that capture the overall size and spatial configuration of each microorganism observed in the image. Among these descriptors are the diameter and area of the outermost circumscribing circle ($d_{ext}$ and $S_{ext}$, respectively), along with the internal area occupied by the microorganism itself ($S_{obj}$).

**Figure 5:**
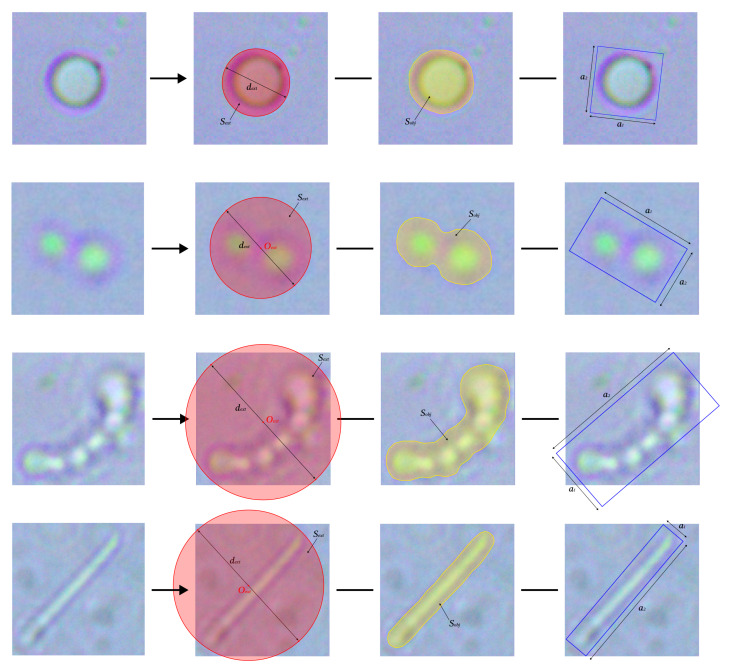
The first category of extracted characteristics of the examined objects. The first line contains features of micrococci, the second of diplococci, the third of streptococci, and the fourth of bacilli.

Further, we compute the dimensions of the smallest rotated rectangle that can fully enclose the object, denoted by its side lengths $a_{1}$ and $a_{2}$. These and other shape-defining parameters collectively form the foundation of our first feature group. Representative examples from this group are presented in Figure 5 organized by microorganism type: micrococci, diplococci, streptococci, and bacilli.

Beyond individual measurements, we also focus on how different features interact by forming pairwise ratios between logically connected parameters. This results in a derived set, $\beta_{0}$, composed of relational metrics that provide additional insight into the morphological proportions and structural nuances of the analyzed samples:

(5) $${\beta_{0}}_{0}=\frac{a_{1}}{a_{2}},\;{\beta_{0}}_{1}=\frac{d_{ext}}{a_{1}},\;{\beta_{0}}_{2}=\frac{S_{obj}}{a_{1}\cdot a_{2}},\;{\beta_{0}}_{3}=\frac{S_{ext}}{a_{1}\cdot a_{2}},\;$$

and so on.

The process of distinguishing between various types of microorganisms and isolating their defining traits relies on a comprehensive utilization of the features contained within the $\beta_{0}$ set. These features are not limited to raw ratio values ${\beta_{0}}_{i}$ derived from earlier geometric measurements but are further enriched through transformation and combination techniques.

Specifically, enhanced descriptors can be generated by scaling individual ratios with empirically determined coefficients ${\alpha_{0}}_{i}$ or by constructing more complex representations through weighted linear aggregations. These aggregations take the general form

$$\sum {\gamma_{0}}_{j}\sum {\alpha_{0}}_{i}^{j}{\beta_{0}}_{i}^{j}*1_{A_{k}}\left(j\right),$$

where ${\gamma_{0}}_{j}$ defines optimization-derived weights obtained during training, and $1_{A_{k}}\left(j\right)$ is an indicator function defining specific feature groupings $A_{k}$ within the power set $2^{\left\{1,...,i*j\right\}}$.

The second category. This stage of the analysis focuses on unveiling deeper and less immediately apparent spatial features of the microorganism, expanding beyond the more basic geometric descriptors discussed earlier. A central aspect involves evaluating the variation in distances from the object’s centroid to its boundary, specifically by measuring both the farthest ($L_{max}$) and nearest ($L_{min}$) contour points relative to the center of mass. Additionally, we consider the radius of the smallest circle that fully encompasses the object ($r_{ext}$), which provides insight into the overall spread of the structure.

To better understand asymmetries and irregularities in object shape, we also calculate the spatial offset between key geometric centers. This includes the distance between the center of mass ($O_{m}$) and the center of the enclosing circle ($O_{ext}$), as well as the separation between $O_{m}$ and the center of the rotated rectangle of maximal area ($O_{recext}$). These spatial relationships help characterize internal imbalances or directional elongation within the microorganism.

**Figure 6:**
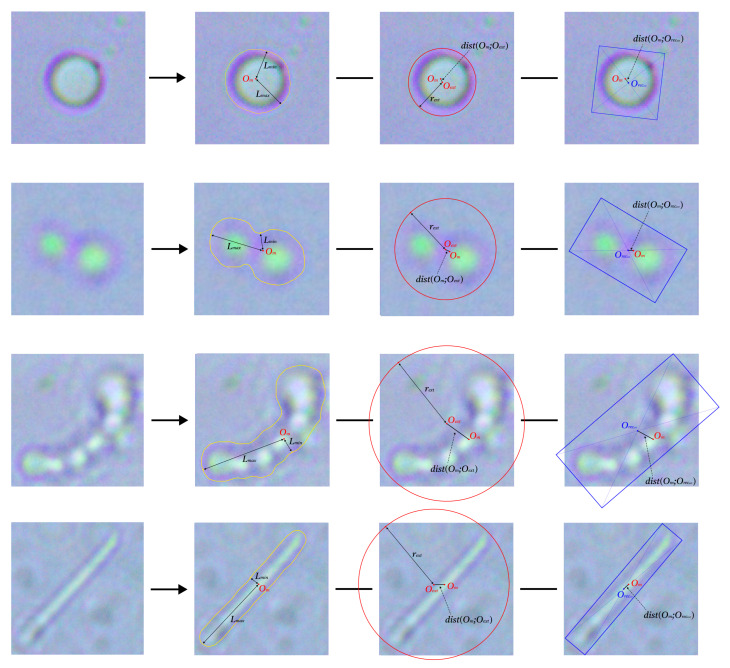
The second category of extracted characteristics of the examined objects. The first line contains features of micrococci, the second of diplococci, the third of streptococci, and the fourth of bacilli.

Figure 6 presents representative visualizations of features belonging to this second category of descriptors.

Building on these measurements, we further construct a feature set $\beta_{1}$ by computing ratios between logically related parameters. Each element of $\beta_{1}$ represents a quantitative relationship that captures interactions between structural dimensions, such as centrality shifts, enclosure tightness, or directional elongation, allowing for a richer and more discriminative morphological representation:

(6) $${\beta_{1}}_{0}=\frac{dist(O_{m};{O_{rec}}_{ext})}{r_{ext}},\;{\beta_{1}}_{1}=\frac{L_{min}}{L_{max}},\;{\beta_{1}}_{2}=\frac{dist(O_{m};O_{ext})}{L_{max}},\;{\beta_{1}}_{3}=\frac{dist(O_{m};O_{ext})}{r_{ext}},\;$$

and so forth.

The feature set $\beta_{1}$ serves as a foundation for distinguishing microscopic structures and reliably classifying various microorganism types. Rather than relying solely on the raw ratios ${\beta_{1}}_{i}$ derived from prior measurements, we expand the representation space by introducing weighted transformations and composite features.

These extended descriptors emerge from two key operations: scaling individual elements ${\beta_{1}}_{i}$ with experimentally determined coefficients ${\alpha_{1}}_{i}$ and constructing hierarchical linear combinations. The latter are defined by

$$\sum {\gamma_{1}}_{j}\sum {\alpha_{1}}_{i}^{j}{\beta_{1}}_{i}^{j}*1_{A_{k}}\left(j\right),$$

where ${\gamma_{1}}_{j}$ defines significant weights optimized during model training, and $1_{A_{k}}\left(j\right)$ is an indicator function that selectively activates feature subsets $A_{k}$ drawn from the power set $2^{\left\{1,...,i*j\right\}}$, with k∈[1..i∗j].

The third category. The third group of descriptors focuses on more complex geometric interactions identified from the microscopic imagery of microorganisms. In particular, we analyze spatial relationships between key geometric centers and boundaries of the object. Notable parameters in this category include the distance from the center of the circumscribed circle ($O_{ext}$) to the center of the largest-area rotated bounding rectangle (${O_{rec}}_{ext}$), denoted as ${K_{1}}_{max}$, and the greatest separation between the object’s outer contour and the edges of this rectangle, referred to as ${K_{1}rec}_{max}$. These values serve as indicators of asymmetry and spatial irregularities within the microorganism’s shape. Additional geometric descriptors complement these measures, offering a more nuanced morphological profile.

**Figure 7:**
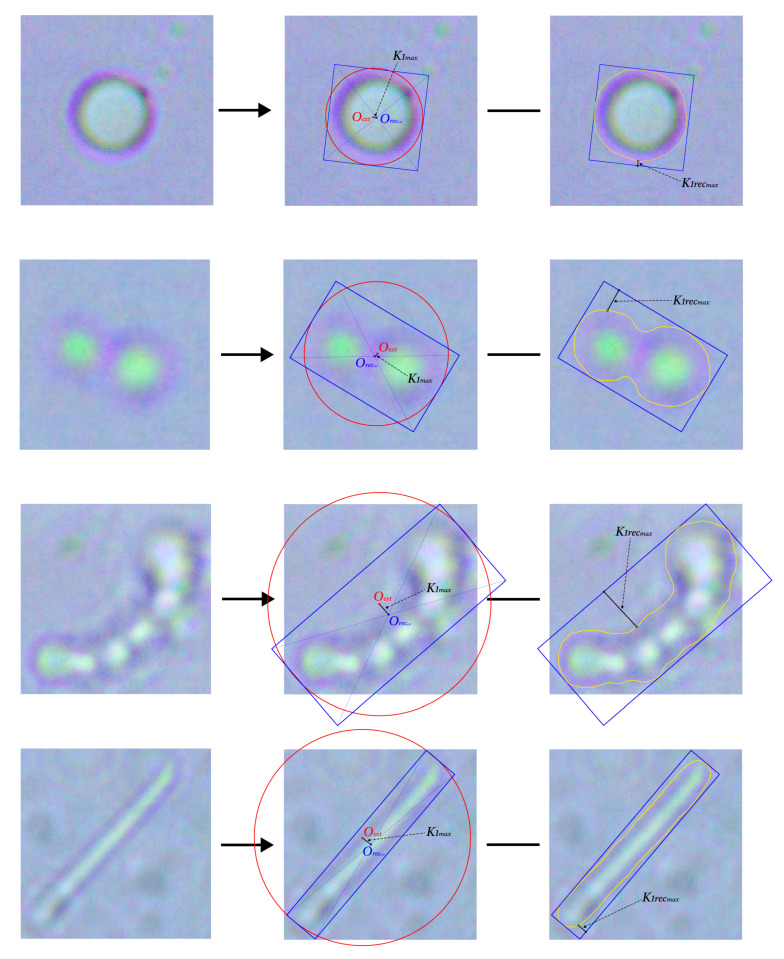
The third category of extracted characteristics of the examined objects. The first line contains features of micrococci, the second of diplococci, the third of streptococci, and the fourth of bacilli.

Representative illustrations of these features are shown in Figure 7.

To further enrich this representation, we examine functionally linked pairs of parameters and compute their ratios to form a derived numerical feature set $\beta_{2}$. Each element in this set captures a structural relationship between two measurements, revealing, for example,

(7) $${\beta_{2}}_{0}=\frac{{K_{1}}_{max}}{{K_{1}rec}_{max}},\;{\beta_{2}}_{1}=\frac{{K_{1}rec}_{max}}{{K_{1}}_{max}},$$

along with other combinations that express complementary dependencies.

The full set $\beta_{2}$ then serves as the basis for identifying the defining features of the observed biological entities and enables accurate classification. Rather than limiting the analysis to raw ratios ${\beta_{2}}_{i}$, we expand the feature space through transformations using empirically selected scaling coefficients ${\alpha_{2}}_{i}$, as well as composite features derived from weighted linear formulations such as

$$\sum {\gamma_{2}}_{j}\sum {\alpha_{2}}_{i}^{j}{\beta_{2}}_{i}^{j}*1_{A_{k}}\left(j\right),$$

where ${\gamma_{2}}_{j}$ defines coefficients optimized during the training phase, and the indicator function $1_{A_{k}}\left(j\right)$ selects active subsets $A_{k}$ within the combinatorial space $2^{\left\{1,...,i*j\right\}}$.

This flexible and layered feature construction framework enables the synthesis of higher-order morphological descriptors by capturing complex dependencies and interactions among geometric attributes. By encoding subtle structural variations and relational patterns, the approach significantly enhances the model’s discriminatory power, improving both the accuracy and robustness of microorganism classification.

### 4.4. Classifiers

For data classification, we employed a range of classifiers commonly utilized in vector space classification tasks. The evaluated methods encompassed Support Vector Machines (SVMs) [65], Linear Regression (LR) [66], Random Forests (RFs) [67], Gradient Boosting Machines (GBMs) [68], and Fully Connected Neural Networks (FCNs) [69]. As demonstrated in the experimental results, the GBM classifier outperformed the others, achieving the highest classification accuracy.

## 5. Experiments, Results, and Discussion

### 5.1. Dataset Description

**Figure 8:**
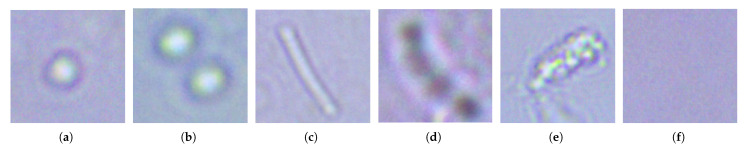
Examples of images from the six classes: (**a**) an image of micrococci; (**b**) an image of diplococci; (**c**) an image of bacilli; (**d**) an image of streptococci; (**e**) an image of another microorganism; (**f**) an image of a random region of microscopy scene that does contain not any microorganism.

The dataset used in this study consists of ~35,000 microscopic images categorized into six distinct classes: micrococci, diplococci, bacilli, streptococci, other microorganisms, and random microscopy regions without bacteria. We used a 5:1:1 split for training, validation, and test sets, respectively. Our dataset was initially balanced; we also used augmentation by rotation and brightness change within acceptable values, which increased the sample size by eight times. These categories reflect both clinically significant bacterial groups and background noise, ensuring robust model generalization. Examples from each class can be found in Figure 8.

All microorganism samples used in this study were obtained exclusively from swab specimens collected from commercially available food products. The dataset comprises biological material extracted from open-access food items representing a broad range of product types found in the consumer market. This approach ensured that the dataset reflects realistic conditions relevant to public health and food safety while avoiding the use of any restricted, clinical, or proprietary sources.

The original images were acquired using a Levenhuk MED D30T microscope, capturing live, unfixed bacterial samples under realistic clinical conditions. The images were carefully annotated and split into three subsets: a training set for model learning, a validation set for hyperparameter tuning, and a test set for final performance evaluation.

This dataset has been made publicly available as an open-access microscopy dataset for microorganism classification. By providing a well-annotated and diverse dataset, we aim to facilitate further research in biomedical image processing, machine learning applications, and rapid pathogen identification.

### 5.2. Experiments

Analyzing the values of ratios and their linear combinations enables the identification of key characteristics of microscopic objects. This plays a crucial role in the development of advanced machine learning models that can accurately detect and classify microorganisms in images. The performance of the models and their respective configurations was systematically evaluated using the following metrics across a set of classifiers:

(8) $$Precision=\frac{TP}{TP+FP},$$

(9) $$Recall=\frac{TP}{TP+FN},$$

(10) $$F_{1}=\frac{2\times Precision\times Recall}{Precision+Recall}.$$

**Figure 9:**
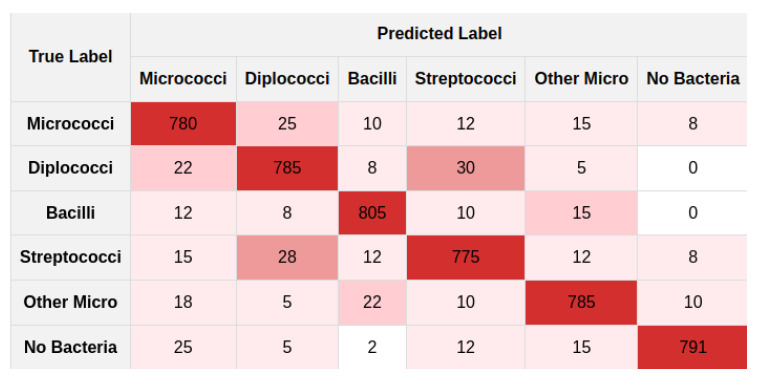
Non-normalized confusion matrix.

**Figure 10:**
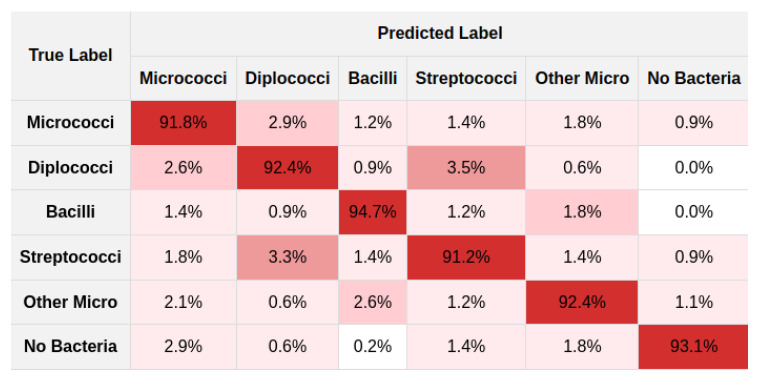
Normalized confusion matrix.

Furthermore, an ablation study was conducted to assess the impact of various filter combinations within the LFIEM framework [60], which was trained on our dataset. The study focused on reconstructing the original image from its distorted counterpart and optimizing the preprocessing pipeline within our integrated classifier scheme. Our preprocessing filter-based module made a significant contribution to overall classifier performance. It should be noted that for training deep classifier models, we used the following environment configuration: an NVIDIA RTX 4090 GPU with 24 GB GDDR6X VRAM paired with an AMD Ryzen 9 7950X processor (16 cores, 32 threads, base clock 4.5 GHz, boost up to 5.7 GHz), 128 GB DDR5 RAM, and a 2 TB NVMe SSD (Samsung 990 Pro), where the models were trained images with RandAugment [70], Mixup [71], CutMix [72], and LFIEM-based preprocessing, using mixed precision [73] and distributed training, optimized via AdamW [74], with an initial learning rate of $3\times 10^{-4}$, weight decay of 0.05, and cosine annealing scheduler [75] over 100 epochs, incorporating label smoothing [76], gradient clipping [77], and early stopping [78] for robust convergence. It should be noted that the method we proposed works in general equally effectively for all types of presented microorganisms, which was confirmed by the corresponding values in the confusion matrices (Figure 9 and Figure 10).

In addition to comparing our method with other approaches, we investigated various configurations of our preprocessing pipeline using the filters from the original paper. The results are presented in Table 1. The best configuration for our method (with Exposure + Contrast + Sharpness filters) is presented along with other best configurations of other methods in Table 2. The highest-performing configurations are summarized in Table 2 as well.

### 5.3. Discussion

The proposed approach for microbial typing demonstrates significant advantages, particularly its ability to analyze non-fixed specimens through specialized preprocessing while maintaining high classification performance metrics. Moreover, our analysis method is quite lightweight and resource-efficient compared to modern deep learning approaches. Our method enables robust morphological feature extraction and ensures interpretability and transparency throughout the classification pipeline. However, the current implementation exhibits certain limitations regarding feature universality and generation procedures, especially when considering the vast diversity of microbial morphologies. Moreover, when analyzing the outlayers, we found out that the main reason was the improper calibration of the capture device integrated with the microscope; further work is planned to be devoted to correcting this shortcoming of the overall system. Also, future research directions should focus on developing automated machine learning (AutoML) procedures for feature generation and the identification of more generalized morphological descriptors to enhance the method’s applicability across broader microbial taxa. Based on the solution of the described problems, a system can be built on the basis of our analytical core in conjunction with the corresponding hardware equipment, solving the problems of automated laboratory analysis of swabs.

**Figure 11:**
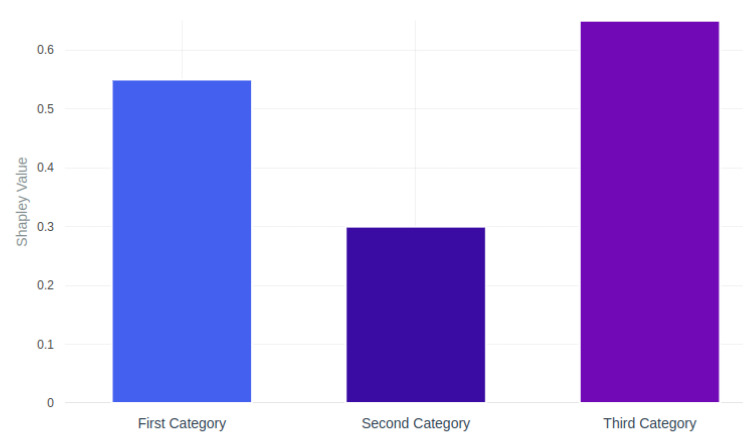
Feature contribution analysis. Mean Shapley values by feature categories.

It should be noted that for our model, all three groups of features had a large contribution to the classification, which was confirmed by the criteria of the mean Shapley values [79] by feature groups (Figure 11).

**Figure 12:**
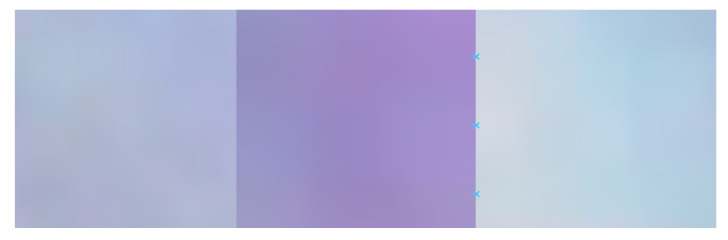
Examples of unsuccessful microscopic scene captures.

Also, when analyzing the errors of our classifier, we found that the largest group was characterized by unsuccessful frame capture by the microscope camera, that is, incorrect calibration parameters of the microscope camera; this problem often cannot be solved using our preprocessing due to very strong distortions or the absence of the necessary information in the frame (Figure 12). As a result, after preprocessing, these images (Figure 12) were classified as missing, but in fact, after calibrating the microscope camera, micrococci were present in this region. That is, errors mostly occurred when other settings of the internal calibration of the microscope camera were required to display visual signs of a microorganism. Even though our dataset is balanced and the quality metrics are high, the automation procedure for such studies should also include a change in the parameters of the internal calibration of the microscope camera, since various properties of the substance being studied are affected differently depending on them.

To balance the dataset and expand the dataset, it is possible to use image transformations (rotations, brightness changes, etc.) that do not change the shape of microorganisms.

## 6. Conclusions

In this study, we developed a hybrid neural network framework tailored for the classification of unfixed microscopic images containing micrococci, diplococci, streptococci, and bacilli. To rigorously assess the effectiveness of our approach, we curated, annotated, and publicly released a specialized dataset. By leveraging explicitly defined, interpretable taxonomic features, our method generated highly distinctive image descriptors, achieving superior performance compared to existing approaches on the evaluated dataset. A notable advantage of our framework is its lightweight architecture: it operates with significantly fewer parameters than typical deep learning models, enabling fast and efficient inference even on standard CPU hardware, making it suitable for real-time applications and deployment in low-resource environments. Additionally, our pipeline facilitated the identification of a set of interpretable taxonomic features, detailed within this work, that can be employed independently of the classifier for manual microbial identification in microscopic images. In future research, we plan to expand this methodology to include additional microbial species and explore its applicability in complex and dynamic microscopic environments.

## Figures and Tables

**Table 1:** Comparison of preprocessing methods with Features Gen + GBM classifier.

Preprocessing Method Combination	Precision	Recall	F1-Score
No preprocessing	0.812	0.803	0.807
Exposure only	0.845	0.832	0.838
Sharpness only	0.827	0.818	0.822
Contrast only	0.851	0.842	0.846
Linear transformation only	0.836	0.824	0.830
Trainable kernel only	0.838	0.829	0.833
Exposure + Sharpness	0.867	0.854	0.860
Exposure + Contrast	0.882	0.871	0.876
Sharpness + Contrast	0.875	0.863	0.869
Blur + Contrast	0.841	0.833	0.837
Linear transformation + Sharpness	0.853	0.841	0.847
Trainable kernel + Exposure	0.864	0.853	0.858
Exposure + Contrast + Blur	0.878	0.866	0.872
Exposure + Linear transformation + Sharpness	0.881	0.870	0.875
Exposure + Contrast + Sharpness	0.910	0.901	0.905
Exposure + Contrast + Sharpness + Blur	0.892	0.881	0.886
All filters combined	0.885	0.874	0.879

**Table 2:** Comparative analysis of classification pipelines using the MMICD dataset (top-30).

Filters Configuration	Classifier Model	Params (M)	FLOPs (G)	Precision	Recall	F1
Exposure + Contrast + Sharpness	MobileNetV3	5.4	0.22	0.723	0.698	0.710
Exposure + Contrast + Sharpness	InceptionResNetV1	27.9	5.71	0.735	0.712	0.723
Exposure + Contrast	ResNet152	60.2	11.31	0.748	0.725	0.736
Exposure + Contrast	EfficientNetB0	5.3	0.39	0.752	0.731	0.741
Exposure + Contrast	Features Gen + SVM	0.8	0.05	0.761	0.739	0.750
Exposure + Contrast	InceptionResNetV2	55.9	12.98	0.768	0.745	0.756
Exposure + Contrast + Sharpness	Features Gen + SVM	0.8	0.05	0.774	0.752	0.763
Exposure + Contrast + Sharpness	EfficientNetB0	5.3	0.39	0.781	0.760	0.770
Exposure + Contrast	ResNet101	44.6	7.85	0.785	0.764	0.774
Exposure + Contrast + Sharpness	EfficientNetB1	7.8	0.70	0.792	0.771	0.781
Exposure + Contrast	EfficientNetB2	9.2	1.01	0.798	0.778	0.788
Exposure + Contrast + Sharpness	ResNet101	44.6	7.85	0.803	0.784	0.793
Exposure + Contrast + Sharpness	EfficientNetB3	12.2	1.86	0.809	0.790	0.799
Exposure + Contrast	EfficientNetB4	19.3	3.39	0.815	0.796	0.805
Exposure + Contrast	CoAtNet	42.1	6.52	0.821	0.803	0.812
Exposure + Contrast	EfficientNetB6	43.0	10.34	0.827	0.810	0.818
Exposure + Contrast	Features Gen + RF	1.2	0.08	0.832	0.815	0.823
Exposure + Contrast	SE-ResNext50	27.6	4.25	0.838	0.821	0.829
Exposure + Contrast + Sharpness	ResNet152	60.2	11.31	0.843	0.827	0.835
Exposure + Contrast + Sharpness	Features Gen + RF	1.2	0.08	0.849	0.833	0.841
Exposure + Contrast	Features Gen + GBM	1.5	0.12	0.854	0.839	0.846
Exposure + Contrast + Sharpness	CoAtNet	42.1	6.52	0.860	0.845	0.852
Exposure + Contrast	ViT-L/16	304.3	190.7	0.866	0.852	0.859
Exposure + Contrast	EfficientNetB3	12.2	1.86	0.872	0.858	0.865
Exposure + Contrast + Sharpness	EfficientNetB4	19.3	3.39	0.878	0.865	0.871
Exposure + Contrast + Sharpness	InceptionResNetV2	55.9	12.98	0.884	0.871	0.877
Exposure + Contrast + Sharpness	SE-ResNext50	27.6	4.25	0.890	0.878	0.884
Exposure + Contrast + Sharpness	ViT-L/16	304.3	190.7	0.896	0.885	0.890
Exposure + Contrast + Sharpness	EfficientNetB6	43.0	10.34	0.902	0.892	0.897
Exposure + Contrast + Sharpness	Features Gen + AutoML	1.8	0.15	0.910	0.901	0.905

Note: Params = Number of trainable parameters in millions (M), FLOPs = Floating Point Operations per inference in billions (G). Our method achieved superior performance with significantly lower computational requirements.

## Data Availability

The dataset collected and used during this study has been made publicly available at the following link: https://github.com/itmo-cv-lab/mmicd (accessed on 1 May 2025).
